# Clinical effects of TP-based hyperthermic intraperitoneal chemotherapy (HIPEC) on CD133 and HE4 expression in advanced epithelial ovarian cancer

**DOI:** 10.12669/pjms.38.6.5287

**Published:** 2022

**Authors:** Xianhui Su, Xuewen Sun, Ying Wang, Yanhui Kang, Yuna Dai

**Affiliations:** 1 Xianhui Su, Department of Pharmacology, Medical College of Hebei Engineering University, Handan 056038, Hebei, China; 2 Xuewen Sun, Department of Pathogenic Biology, Medical College of Hebei Engineering University, Handan 056038, Hebei, China; 3 Ying Wang, Department of Pharmacology, Medical College of Hebei Engineering University, Handan 056038, Hebei, China; 4 Yanhui Kang, Department of Pharmacology, Medical College of Hebei Engineering University, Handan 056038, Hebei, China; 5 Yuna Dai, Department of Breast Surgery, Affiliated Hospital of Hebei Engineering University, Handan 056029, Hebei, China

**Keywords:** Advanced epithelial ovarian cancer, Intraperitoneal chemotherapy, TP regimen, Antigen cluster protein 133 (CD133), Human epididymal secretory protein 4 (HE4)

## Abstract

**Objectives:**

To investigate the clinical effects of TP-based hyperthermic intraperitoneal chemotherapy (HIPEC) on the levels of antigen cluster protein 133 (CD133) and human epididymal secretory protein 4 (HE4) in patients with advanced epithelial ovarian cancer (EOC).

**Methods:**

A total of 104 patients with advanced EOC hospitalized in Affiliated Hospital of Hebei Engineering University from April 2015 to December 2018 were assigned to two groups using a random number table. A control group (n =52) treated by the conventional postoperative TP regimen and an observation group (n =52) receiving HIPEC in addition to the conventional postoperative TP regimen. CD133 and HE4 expression in serum, overall response rate (ORR), long-term efficacy, and incidence of drug toxicity were measured for comparative analysis.

**Results:**

The serum levels of CD133 and HE4 expression in the observation group were lower than in the control group (P < 0.005, respectively); the observation group surpassed the control group in ORR, 2-year survival, and progression-free survival (PFS) (P < 0.005, respectively); however, the two groups had no statistically significant difference in the incidence of drug toxicity (P > 0.05).

**Conclusions:**

TP-based HIPEC can effectively inhibit CD133 and HE4 expression in advanced EOC, which thereby improves the clinical efficacy and encourages longer survival.

## INTRODUCTION

Ovarian cancer is one of the three major types of malignancies that threaten women’s health^1^. Epithelial ovarian cancer (EOC) is the most common type of ovarian cancer, accounting for about 90% of all cases.^2^ Cytoreductive surgery (CRS) and subsequent TP induction chemotherapy are accepted as standard treatments for EOC.^3^,^4^ TP regimen refers to the combination therapy of paclitaxel chemotherapy drugs and platinum drugs, where T stands for paclitaxel drugs and P refers to the code name containing platinum drugs. However, current treatment outcomes are far from satisfactory among EOC patients as the 5-year survival is relatively low mainly because postoperative implantation metastasis from EOC into the abdominal cavity occurs in over 50% of all cases.^5^ Hyperthermic intraperitoneal chemotherapy (HIPEC) is a new treatment option that allows for efficient delivery of heated chemotherapy drugs into the abdominal cavity to achieve high local concentrations of anticancer drugs.

Therefore, it is a preferred procedure to eradicate intraperitoneal free cancer cells or residual cancer foci.^6^ On this basis, a comparative analysis was conducted in this study to assess the clinical efficacy of the conventional TP regimen and TP-based HIPEC in treating EOC patients and further validate the clinical value of HIPEC. Additionally, the expression levels of the cancer stem cell marker antigen cluster protein 133 (CD133) and the tumor biomarker human epididymal secretory protein 4 (HE4) in serum were measured to provide a reference for the interpretation of HIPEC’s clinical efficacy in patients with EOC.

## METHODS

### Demographic and clinical data

This study included 104 patients who were diagnosed with advanced EOC and admitted by our hospital for treatment during April 2015 and December 2018. The patients were randomized into a control group (subject to conventional postoperative TP chemotherapy) and an observation group (receiving conventional postoperative TP chemotherapy and HIPEC) using a random number table, with each group having 52 patients. The control group was at the mean age of (51.5 ±12.4) years; of the 52 patients, 16 were diagnosed with Stage IIIB EOC, 25 with Stage IIIC EOC, and the rest 11 with Stage IV EOC; pathologically, there were 29 cases of serous cystadenoma, 19 of mucinous cystadenoma, 2 of endometrioid carcinoma, and 2 of undifferentiated carcinoma. In the observation group, the mean age was (53.4 ± 12.1) years; Among the 52 patients, 17 had Stage IIIB EOC, 25 had Stage IIIC EOC, and 10 had Stage IV EOC; pathologically, there were 27 cases of serous cystadenoma, 20 of mucinous cystadenoma, 3 of endometrioid carcinoma, and 2 of undifferentiated carcinoma. Differences between the two groups in age, clinical staging, and pathological classification lacked statistical significance (P > 0.05).

### Ethical Approval

The study was approved by the Institutional Ethics Committee of Affiliated Hospital of Hebei Engineering University on April 10, 2016(No. [2016]028), and written informed consent was obtained from all participants

### Inclusion criteria

• A patient was rendered eligible for the study if he/she was:
• Confirmed to have ovarian cancer based on pathological diagnosis;
• Underwent CRS;
• Met the criteria for FIGO stage IIIB-IV ovarian cancer^7^;
• Had an expected survival of at least 3 months.

### Exclusion criteria

• A patient was excluded if he/she met any of the following criteria:
• Having a history of radiotherapy, chemotherapy or surgical treatment in the past three months;
• Complicated with severe dysfunction of vital organs (e.g., liver, kidney, etc.).

All patients gave informed consent for the study. The control group was given conventional TP chemotherapy following CRS: weekly TP regimen with 135 mg/m² paclitaxel (PTX) (Hisun-Pfizer Pharmaceuticals Co., Ltd., H20059378) via intravenous drip infusion on D1 and 75 mg/m² cisplatin (CP) (Qilu Pharmaceutical (Hainan) Co., Ltd., H20073653) via intravenous drip infusion on D2. The observation group underwent HIPEC as an add-on to the conventional TP regimen. In other words, 135 mg/m² PTX was dissolved in 5% dextrose solution for HIPEC, followed by delivery of 60 mg/m² CP in 0.9% physiological salt solution via intravenous infusion on the other day. During HIPEC, each patient was instructed to adjust their positions to ensure homogeneous distribution of chemotherapy drugs in the abdominal cavity. The TP regimen was administered at regular intervals for two weeks after CRS. The treatments were repeated every 21 days for three cycles. Clinical efficacy was evaluated while relevant serological markers were measured at the end of the treatment program.

### Outcome measures

Serum CD133 and HE4 expression: Fasting venous blood samples (5 mL/each) were collected from each patient before and after 3 cycles of treatment. Blood serum was isolated from each sample via centrifugation after standing for 30 min at room temperature. Subsequently, the serum levels of CD133 and HE4 were determined using an ELISA assay kit.

Short-term efficacy: According to RECIST 1.1,^8^ tumor response was classified into four categories, including complete response (CR), partial response (PR), stable disease (SD), and progressive disease (PD). ORR = (number of CR patients + number of PR patients) / total number of patients ×100%.

### Adverse reactions

Toxic side effects of chemotherapy drugs were evaluated following the NCI Common Terminology Criteria for Adverse Events v3.0 (CTCAE v3.0).^9^ 4) Survival analysis: Phone calls and regular outpatient visits (every 3 months) were made to follow up on patients, and the median follow-up was (25.2 ±4.2) months. A PFS curve was plotted by calculating the one sand two years survival. Disease-free survival (DFS) is defined as the time from commencement of treatment to disease progression or death.

### Statistical Analysis

The software SPSS25.0 was used for data analysis. Enumeration data were represented by the number or percentage of patients [n(%)] and analyzed by the Χ^2^ test; measurement data were expressed as ‘((±s)’ and examined by the independent samples t-test. The Kaplan-Meier method was used for survival analysis. Results were considered significant if P < 0.05.

## RESULTS

Differences in the serum levels of CD133 and HE4 between the two groups were not significant before treatment (P > 0.05); at the end of the treatment program, the serum levels of CD133 and HE4 in both groups were significantly reduced in comparison with the pre-treatment levels (P < 0.05), and the observation group exhibited sharper decreases in serum CD133 and HE4 compared with the control group, with the differences demonstrating statistical significance (P < 0.05) Table-I.

**Table I:** Comparison of Serum CD133 and HE4 Between the Two Groups (*x̅*±s).

Group	CD133 (U/mL)				HE4 (pmol/L)			

	Pre-treatment	Post-treatment	*t*	*P*	Pre-treatment	Post-treatment	*t*	*P*
Observation (n =52)	135.4±18.4	29.3±5.5	39.840	0.000	228.1±41.9	39.7±10.5	31.452	0.000
Control (n =52)	129.5±21.7	74.2±10.3	16.601	0.000	231.8±44.5	84.2±16.5	22.426	0.000
t	1.495	27.729			0.437	16.408		
P	0.138	0.000			0.663	0.000		

The observation group had an ORR of 75% (39/52), while the control group had an ORR of 55.8% (29/52); the difference between the two groups was statistically significant (Χ^2^ = 4.248, P = 0.039; U =2.600, P = 0.009) Table-II.

**Table II:** Comparison of Clinical Efficacy Between the Two Groups [n (%)].

Group	CR	PR	SD	PD	ORR
Observation (n =52)	9 (17.3)	30 (57.7)	12 (23.1)	1 (1.9)	39 (75.0)
Control (n =52)	4 (7.7)	25 (48.1)	13 (25.0)	10 (19.2)	29 (55.8)

During the treatment program, there were 38 cases of toxic side effects in total, including 21 cases in the observation group and 17 cases in the control group, and the difference between the two groups lacked statistical significance (P > 0.05) Table-III.

**Table III:** Comparison of Incidence of Toxic Side Effects Between the Two Groups [n (%)].

Group	Grade III-IV gastrointestinal toxicity	Grade II-III bone marrow suppression	Neurotoxicity	Overall incidence rate
Observation (n =52)	8 (15.4)	7 (13.5)	6 (11.5)	21 (40.4)
Control (n =52)	5 (9.6)	6 (11.5)	6 (11.5)	17 (32.7)
Χ^2^	0.791	0.088	-	0.663
P	0.373	0.767	-	0.415

### Survival Analysis

**Fig.1:**
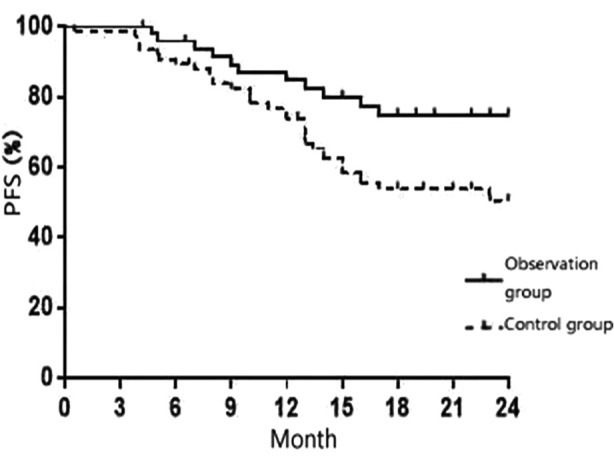
Comparison of PFS Between the Two Groups.

As at the end of the follow-up, there were 10 deaths in the observation group and 20 deaths in the control group. The difference in 1-year survival between the two groups was not significant (88.5% vs 78.9%, Χ^2^ = 1.758, P = 0.185); however, the two group exhibited a statistically significant difference in 2-year survival (80.8% vs 61.5%, Χ^2^ = 4.685, P = 0.034). Additionally, the difference in PFS between the two groups was also statistically significant (Χ^2^ =7.537, P = 0.031) Fig.1.

## DISCUSSION

Currently, the 5-year survival rate for patients with Stage I-II ovarian cancer is up to 70%, and the prognosis is satisfactory overall. However, for those with Stage III ovarian cancer, the 5-year survival rate is merely 10-25%^10^, in which cases intraperitoneal implantation and distant metastasis are two key indicators for progression of advanced ovarian cancer. HIPEC is a novel dosing regimen that effectively reduces the risk of recurrent intraperitoneal implantation after surgery. By reasonably combining the advantages of thermotherapy, chemotherapy, and intraperitoneal infusion, HIPEC improves the clinical efficacy of chemotherapy drugs by delivering the chemotherapy drugs with anticancer activities into the abdominal cavity to directly act on the cancer tissue and creating a high-temperature environment to induce cancer cell apoptosis, enhance capillary permeability and promote absorption of chemotherapy drugs in the blood stream by the cancer tissue.^11^ HIPEC has distinct pharmacokinetic and pharmacodynamic advantages over intravenous chemotherapy as it supports more powerful and long-lasting anticancer effects with sustained high blood concentrations of chemotherapy drugs in the abdominal cavity.^12^ As shown in this study, after three chemotherapy cycles, the clinical efficacy was significantly improved in the observation group compared with the control group, without significant difference in the incidence of adverse reactions between the two groups. Older patients with ovarian cancer are shown to have a higher risk of recurrence because of immune response and drug resistance.^13^ HIPEC also applies to patients with drug-resistant and refractory advanced ovarian cancer.^14^ It is reported^15^ that direct infusion of chemotherapy drugs into the abdominal cavity without metabolism by the liver or transport to the systemic circulation, which therefore produces no additional toxic side effects.

CD133 is currently an extensively used cancer stem cell marker associated with the degree of malignancy, invasion, infiltration and metastasis of cancer cells.^16^,^17^ Given that a high serum level of CD133 expression in ovarian cancer is associated with a poor prognosis, it is considered that high CD133 expression indicates the presence of a large number of cancer stem cells, and CD133^+^ is shown to induce tumor vascularity, thereby promoting cancer cell proliferation and metastasis.^18^-^20^ Additionally, HE4 is demonstrated to be highly expressed in many human cancer tissues, such as cervical cancer, breast cancer, lung cancer, and ovarian cancer, and have direct correlations with the ability of infiltration and migration of cancer cells.^21^ In the meanwhile, it is reported that the combined use of CA125 and HE4 can improve the accuracy of early diagnosis of ovarian cancer and help predict the prognosis and treatment outcomes of patients with ovarian cancer.^22^ Since HE4 has a molecular weight significantly lighter than CA125, it is easier for HE4 to circulate in the bloodstream, which suggests greater specificity and sensitivity for the early diagnosis of ovarian cancer.^23^ Considering that intraperitoneal implantation of cancer cells is correlated with the number, activity, and ability of infiltration and migration of cancer stem cells, this study provided a comparative analysis of the pre- and post-treatment serum levels of CD133 and HE4. The study results revealed that although the serum levels of CD133 and HE4 in both groups following three cycles of treatment were lower than the pre-treatment levels, these markers were reduced at a faster pace in the observation group compared with the control group, and the difference was statistically significant (P < 0.05). Despite the use of PTX and CP in both groups, the observation group achieved better clinical efficacy as HIPEC at 42°C mediated the optimal efficacy of CP, induced cancer cell apoptosis directly, enhanced the membrane permeability of cancer cells and increased the PTX concentration in cancer cells.^7^ This procedure was performed directly in the abdominal cavity, which substantially strengthened the effect of eradicating residual cancer cells and foci after CRS. Further, the follow-up results demonstrated that the observation group outperformed the control group in 2-year survival and DFS in spite of the lack of significant difference in 1-year survival between the two groups. Therefore, it is inferred that 2-year survival and DFS may have correlations with the level of residual cancer stem cells.

### Limitations of this study

The number of subjects included in this study was limited, so the conclusions drawn may not be very convincing. In addition, we only analyzed and discussed the cases included in our hospital, which may not be representative enough. We look forward to a multi-center study in the future to reach more comprehensive conclusions.

## CONCLUSION

TP-based HIPEC can help significantly reduce the serum levels of CD133 and HE4 expression and remarkably improve the clinical efficacy and long-term survival in patients with advanced EOC. Therefore, this regimen is worthy of wider clinical application. However, since this study is limited by its sample size and follow-up period, a larger clinical sample size and an extended follow-up period are needed for future studies to reach more reliable conclusions.

### Authors’ Contributions:

**XS &** **XS:** Designed this study, prepared this manuscript, are responsible, accountable for the accuracy and integrity of the work.

**YW &** **YD:** Collected and analyzed clinical data.

**YK:** Significantly revised this manuscript.
